# Dynamic local metrics changes in patients with toothache: A resting-state functional magnetic resonance imaging study

**DOI:** 10.3389/fneur.2022.1077432

**Published:** 2022-12-12

**Authors:** Mengting Wang, Xin Tang, Bin Li, Tianyi Wan, Xuechao Zhu, Yuping Zhu, Xunfu Lai, Yulin He, Guojin Xia

**Affiliations:** ^1^Medical Imaging Center, The First Affiliated Hospital of Nanchang University, Nanchang, China; ^2^Medical Imaging Center, Jiangxi Provincial People's Hospital, Nanchang, China; ^3^Medical Imaging Center, Jiangxi Cancer Hospital, Nanchang, China

**Keywords:** resting-state functional magnetic resonance, dynamic local metrics, pain, toothache, pathological mechanism

## Abstract

**Objective:**

To study the dynamic changes of local metrics in patients with toothache (TA, Toothache) in the resting state, in order to further understand the changes of central neural mechanism in patients with dental pain and its effect on cognition and emotion.

**Methods:**

Thirty patients with TA and thirty matched healthy (HC) control volunteers were recruited, and resting-state functional magnetic resonance (rs-MRI) scans were performed on all subjects, and data were analyzed to compare group differences in three dynamic local indices: dynamic regional homogeneity (dReHO), dynamic low-frequency fluctuation amplitude (dALFF) and dynamic fractional low-frequency fluctuation amplitude (dfALFF). In addition, the association between dynamic local metrics in different brain regions of TA patients and scores on the Visual Analog Scale (VAS) and the Hospital Anxiety and Depression Scale (HADS) was investigated by Pearson correlation analysis.

**Results:**

In this study, we found that The local metrics of TA patients changed with time Compared with the HC group, TA patients showed increased dReHo values in the left superior temporal gyrus, middle frontal gyrus, precentral gyrus, precuneus, angular gyrus, right superior frontal gyrus, middle temporal gyrus, postcentral gyrus and middle frontal gyrus, increased dALFF values in the right superior frontal gyrus, and increased dfALFF values in the right middle temporal gyrus, middle frontal gyrus and right superior occipital gyrus (*p* < 0.01, cluster level *P* < 0.05). Pearson correlation analysis showed that dReHo values of left precuneus and left angular gyrus were positively correlated with VAS scores in TA group. dReHo value of right posterior central gyrus was positively correlated with HADS score (*P* < 0.05).

**Conclusion:**

There are differences in the patterns of neural activity changes in resting-state brain areas of TA patients, and the brain areas that undergo abnormal changes are mainly pain processing brain areas, emotion processing brain areas and pain cognitive modulation brain areas, which help to reveal their underlying neuropathological mechanisms. In the hope of further understanding its effects on cognition and emotion.

## Introduction

Toothache (TA) is a common pain in the maxillofacial area, which can be divided into odontogenic and non-odontogenic toothache. Odontogenic toothache mainly due to various irritations of the tooth itself or its surrounding soft tissues, such as pulpitis, periodontitis, pericoronitis, periapical inflammation, and cavities. Previous studies have shown that odontogenic toothache is mainly mediated by Toll-like receptors (TLR). Microbial infection or tissue damage releases endogenous ligands, which activates TLR and leads to the up-regulation of NF-κB, which causes a series of downstream reactions and affects peripheral nerve endings, leading to the generation of pain (1). Some non-dental changes can also manifest as toothache, such as temporomandibular joint disorder (TMDS), primary headache, trigeminal neuropathy, and others (2). The neurotransmission mechanism of toothache shows that external stimuli cause sensitization of afferent neurons, and afferent divine stimulation of trigeminal neurons expresses TRPV1, TRPV2, and RPM8; these receptors transduce specific pain stimuli into electrical impulses that stimulate the trigeminal thalamic tract, spinal thalamic tract, reticular thalamic bundles, and finally, through the thalamus, to the cerebral cortex, causing a pain response (3, 4). In daily life, approximately 7–32% of people suffer from TA (5). Studies have shown that psychological factors, dental care, and TA are also closely related (6). Untreated pain may cause certain physiological and pathological reactions, such as increased heart rate and blood pressure due to sympathetic excitation, and prolonged TA affects the patient's sleep, diet, and other daily activities. In serious cases, it can lead to psychological conditions, such as anxiety, irritability, and depression (7, 8). Studies have shown that maxillofacial pain is more prone to anxiety, depression and other conditions during the COVID-19 epidemic (9). In addition, due to the close anatomical location of the periodontal and paranasal sinuses, oral infection of the maxillary posterior teeth and inflammation after root canal treatment can spread to the paranasal sinuses, leading to odontogenic sinusitis, accounting for 60–80% of maxillary sinusitis. Severe sinus infection may lead to cavernous sinus thrombosis, brain abscess, meningitis, subdural abscess and other complications. In severe cases, it can even be life-threatening (10, 11). This can be a great burden for both the clinic and the patient. Studies have shown that TA patients exhibit changes in brain function; however, the differences in dynamic spontaneous brain activity between TA patients and healthy controls remain unknown, and the altered central neural mechanisms of TA and their effects on emotion and cognition are currently unclear.

rs-MRI is a tool to study spontaneous functional alterations in the human brain by measuring changes in blood oxygen level-dependent (BOLD) signals; it is a non-invasive and reproducible tool for studying functional alterations in the brain that is widely used in clinical research (12–14). Studies have shown that TA increases ALFF in the left postcentral gyrus, right paracentral lobule, right lingual gyrus, right inferior occipital gyrus, left syrinx, and right superior occipital gyrus (15), and increases degree centrality (DC) values in the right lingual gyrus, right precentral gyrus, and left middle temporal gyrus (16). Yang et al. (17) showed that the PerAF signal in the right dorsolateral superior frontal gyrus and right postcentral gyrus of patients with TA was lower than that in HC. The above studies provide a basis for neuroimaging to better understand the consciousness and cognitive changes associated with TA. However, the aforementioned local activity characteristics were measured assuming that the BOLD signal was stationary during the fMRI scan, ignoring the dynamic characteristics of spontaneous brain activity over time. Studies have shown that the brain is not a static system but a highly dynamic system that responds to internal and external stimuli by dynamically integrating and adjusting multiple time scales (18–20). Regional homogeneity, low-frequency fluctuation amplitude, and fractional low-frequency fluctuation amplitude are all valid reflections of the local features of brain function (21). Regional homogeneity (ReHo) (22), which primarily measures the temporal synchronization of regional neural activity between spatially adjacent regions. Both low-frequency fluctuation amplitude (ALFF) (23) and fractional low-frequency fluctuation amplitude (fALFF) (24) can reflect the intensity of regional neural activity. ALFF measures the signal intensity in low-frequency oscillations of local spontaneous neural activity, and fALFF measures the ratio of the amplitude in the low-frequency range to the total amplitude in the full frequency range, revealing the relative contribution of a specific ALFF to the entire frequency range. These three local features reveal different aspects of the altered local brain activity (21). However, all of these can only reflect the stationary characteristics of brain activity, which contradicts the idea that the brain is a highly dynamic system. Therefore, an increasing number of studies have focused on dynamic changes in brain activity. Dynamic Regional homogeneity (dReHo), dynamic low-frequency low-frequency fluctuation amplitude (dALFF), and dynamic fractional low-frequency fluctuation amplitude (dfALFF) have been used to study the dynamic characteristics of spontaneous brain activity in various diseases. Dynamic local indicators reflect the magnitude of changes in the brain over time better than static local indicators and are complementary to many static local indicators (25, 26).

This study was based on a sliding window (27, 28) to study the dynamics of local metrics in patients with TA. We hypothesize that changes in local indicators over time are more pronounced in TA patients than in healthy controls. The aim is to provide more information to study the neuropathological mechanisms of TA and the effects on emotion and cognition.

## Materials and methods

Based on the Declaration of Helsinki, This study was approved by the Institutional Review Board of the First Affiliated Hospital of Nanchang University. Written informed consent was obtained from each subject before the study.

### Participants

Thirty TA patients were recruited from the Department of Dentistry, The First Affiliated Hospital of Nanchang University, Diagnosis by an experienced dentist. thirty volunteers matched for gender, age, and education level of TA patients were also recruited, all of whom were right-handed.

The inclusion criteria for the TA group were as follows: 1) Pain in the pulp and/or periodontal tissues caused by dental or non-dental diseases; 2) Acute or chronic TA; 3) No other diseases causing painful symptoms (such as postherpetic neuralgia, lower back pain, knee pain, migraine, et al.); 4) Toothache with no obvious etiology that could not be attributed to other diseases; 5) Conventional MRI of the head was normal; 6) All subjects had no contraindications to MRI examination (such as such as metal implants or claustrophobia, et al.); 7) Absence of psychiatric and neurological disorders (such as neurodegenerative diseases, epilepsy, Alzheimer's disease, bipolar disorder, mania, depression, and Schizophrenia et. al.), cardiovascular disease, cerebral infarction, diabetes, hypertension, et al.

The exclusion criteria for the TA group were as follows: 1) Presence of non-toothache; 2) First-degree relatives with hereditary non-toothache syndrome; 3) Conventional MRI scan with functional and structural lesions in the brain; 4) History of head and facial trauma and surgery; 5) Contraindications for MRI, such as metal implants or claustrophobia; 6) Psychiatric and neurological disorders, cardiovascular diseases, cerebral infarction, diabetes, hypertension, et al.; 7) Incomplete data from MRI scans or clinical assessment.

Inclusion criteria for 30 healthy controls: 1) No symptoms of odontogenic and non-odontogenic toothache; 2) Conventional MRI of the head was normal; 3) Absence of psychiatric and neurological disorders, cardiovascular disease, cerebral infarction, diabetes, hypertension, et al.; 4) Denied a history of drug or alcohol abuse; 5) No other diseases causing painful symptoms.

### Clinical and cognitive psychological assessments

Prior to the MRI scan, the investigators collected clinical information from the subjects, including gender, age, duration of toothache, duration, pain location, past history, and HADS score. The pain level was assessed using a VAS. A 10-cm ruler was used to score the subject's pain from 0 to 10, and a scale of 0–10 was marked on the ruler for the patient to mark the level of pain on the ruler. The higher the score, the greater the intensity of the pain. A score of “0” indicates no pain and “10” indicates severe and intolerable pain. The cognitive function of patients were assessed by MoCA (Montreal Cognitive Assessment Scale).

### Resting-state functional magnetic resonance imaging data acquisition

All participants used a 3.0T magnetic resonance system (Siemens, Erlangen, Germany) scanner with 8-channel phased-array magnetic head coils to collect MRI data in the First Affiliated Hospital of Nanchang University. The subjects were informed of the precautions to be taken and their cooperation was obtained before the scans. During the scans, the subjects were placed supine on the examination bed, head first, The head was immobilized with foam and the subjects were given noise-canceling headphones to reduce head movements and noise effects. During the examination, all subjects were awake and had their eyes closed. Prior to the resting-state functional MRI scans, all subjects underwent routine T1-weighted and T2-weighted image acquisition to exclude many structural brain lesions that could affect brain function and microstructure. Resting-state functional MRI scans included 176 T1 structural images and 240 rs-fRMI functional images.T1 structural images were scanned with *TE* = 2.26 ms, *TR* = 1,900 ms, flip angle = 9°, acquisition matrix = 256 × 256, field of view = 250 × 250 mm, thickness = 1.0 mm, gap = 0.5 mm, voxel 1.0 × 1.0 × 1.0 mm. rs-fRMI functional image scan parameters *TE* = 30 ms, TR = 2,000 ms, flip angle = 90°, acquisition matrix = 64 × 64, field of view = 220 × 220 mm, thickness = 4.0 mm, gap = 1.2 mm, voxel 3.0 × 3.0 × 4.0 mm.

At the end of the scan, the image quality should be checked, and if the image does not meet the requirements, the results of this scan should be discarded, or the scan should be repeated with the subject's permission.

### Data preprocessing

The scanned completed data were analyzed using MRIcro software (http://www.MRIcro.com) to analyze the acquired information and exclude incomplete data. Preprocessing of resting-state functional MR images and structural images was performed in the SPM12 (http://www.fil.ion.ucl.ac.uk/spm) and DPABI packages (29)(http://rfmri.org/DPABI)(http://rfmri.org/DPABI) running on Matlab R2018b. The preprocessing steps were as follows: The DICOM format was converted to NIFTI by removing the first 10 time points for each participant to eliminate the effects of magnetic saturation and MRI scanner noise; then time-layer correction and head motion correction for the remaining 230 volumes (30), during the fMRI scans, subjects had no more than 2.0 cm of maximum displacement in any direction and no more than 2.0° of angular rotation in any axial position, of which three TA patients were excluded. The resultant maps after the conversion format were then segmented using the segmentation method in SPM12, and the spatially normalized functional images of subjects in MNI were obtained using a nonlinear transformation procedure to spatially normalize all functional data to the Montreal Neurological Institute (MNI) template with a voxel size resampling of 3 × 3 × mm (31); data for which dALFF and dfALFF calculations were performed were smoothed with a 4 mm FWHM filtering for smoothing, and band-pass filtering (0.01–0.08 Hz) was used for dReHo calculations to reduce the effects of low-frequency drift, physiological high-frequency noise, and heart noise.Finally covariates were removed, including head motion parameters, mean white matter signal, and cerebrospinal fluid signal.

### Dynamic data processing

Dynamic local metrics analysis is performed using the time-dynamic analysis (TDA) toolkit based on DPABI (32). For dynamic metrics, the most important thing is the choice of the window length, which should be short enough to capture transient signals but long enough to detect slowly changing signals (33, 34). To avoid introducing spurious fluctuations, the minimum window length should be greater than 1/fmin, where fmin is the minimum frequency of the time series (35). Currently, there is no uniform standard for the choice of window length. In this study, a sliding window length of 30 TR (60 s) and a moving step of 2 TR (4 s) were used to split the time course into a 60 s Hamming window with 101 overlapping windows (26, 36). Using this sliding window, dReHO, dALFF, and dfALFF were calculated. dReHO was calculated as the KCC value between the core voxels and the other 26 neighboring voxels. dReHO was calculated after calculating the ReHo for all voxels in the time window, and each participant received several window-based ReHO plots. We then calculated the mean and standard deviation (SD) of each voxel in all window-based ReHO plots for each participant and obtained the corresponding coefficient of variation (CV = SD/mean). Finally, the CV maps were spatially smoothed using an isotropic Gaussian kernel with a FWHM of 4 mm. Spatially smoothed CV maps were used for further statistical analysis. After calculating ALFF, fALFF of all voxels in time windows, each participant will get several window-based ALFF maps, fALFF maps. Then, we computed the mean and SD of each voxel in all window-based ALFF maps, fALFF maps for each participant and further got the CV. The CV maps were prepared for further statistical analysis.

### Statistical analyzes

To detect group differences in demographic variables between TAs and HCs, two-sample *t*-tests and chi-square analyses were performed using statistical software (SPSS 20.0; SPSS, Chicago, IL, USA). Age was compared between patients with TA and HCs using a two-sample *t*-test. Sex differences were determined using chi-square tests.

Statistical comparisons of dReHO, dAFLL, and dfALFF in the HC and TA groups were performed using two-sample two-sided *t*-tests in the SPM12 software (corrected with GRF, two-tailed, *P* < 0.01; voxel level, *P* < 0.05; cluster level, with head movement, age, and sex as covariates).

### Validation analysis

To further test the reliability of our results for the three dynamic local metrics, we reanalyzed the rs-fMRI data using two additional window lengths (20 and 40 TRs).

### Correlation analysis

Dpabi Viewer software was used to obtain the locations of the brain regions for statistical analysis. The dReHO, dAFLL, and dfALFF values of the abnormal brain regions in the patient group were extracted, and Pearson linear analyses of dReHO, dAFLL, and dfALFF values with VAS and HADS scores were performed using SPSS Statistics (version 20.0; *P* < 0.05).

## Results

### Clinical data

There was no statistically significant difference in age and sex between the TA and HC groups (*P* = 0.067; *P* = 0.787), with ages of 26.06 ± 4.85 and 28.20 ± 3.95, respectively (Table 1).

**Table 1:** Demographics and behavioral results of TA and HC groups.

	**TA (*n* = 30)**	**HC (*n* = 30)**	***T*-value**	***P*-value**
Age (year, mean ± SD)	26.06 ± 4.85	28.20 ± 3.95	0.156	0.067
Gender (male/female)	10/20	11/19	N/A	0.787
Leigh hand (right/Left)	30/0	30/0	N/A	-
Duration of illness (days)	2.71 ± 1.09	-	N/A	-
VAS score (mean ± SD)	5.33 ± 1.58	-	N/A	-

Independent *t*-tests comparing the two groups (*p* < 0.05 represented statistically significant differences). Data shown as mean standard deviation or n. TA, toothache; HC, healthy control; N/A, not applicable; VAS, Visual Analog Scale.

### Differences in d-ReHo, d-ALFF, and d-fALFF

**Figure 1:**
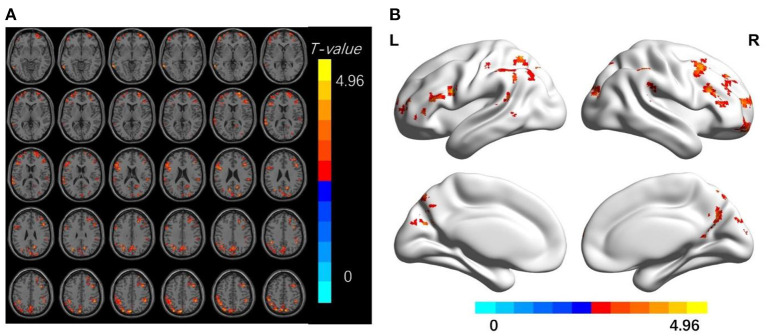
**(A,B)** The differences in dReHo between the TA patients and HCs (after GRF correction voxel-wise *p* < 0.01, cluster-wise *p* < 0.05, two-tailed). The color bar indicates the *t*-value. d-ReHo, dynamic regional homogeneity; HCs, healthy controls; TA, toothache; MNI, Montreal Neurological Institute.

**Figure 2:**
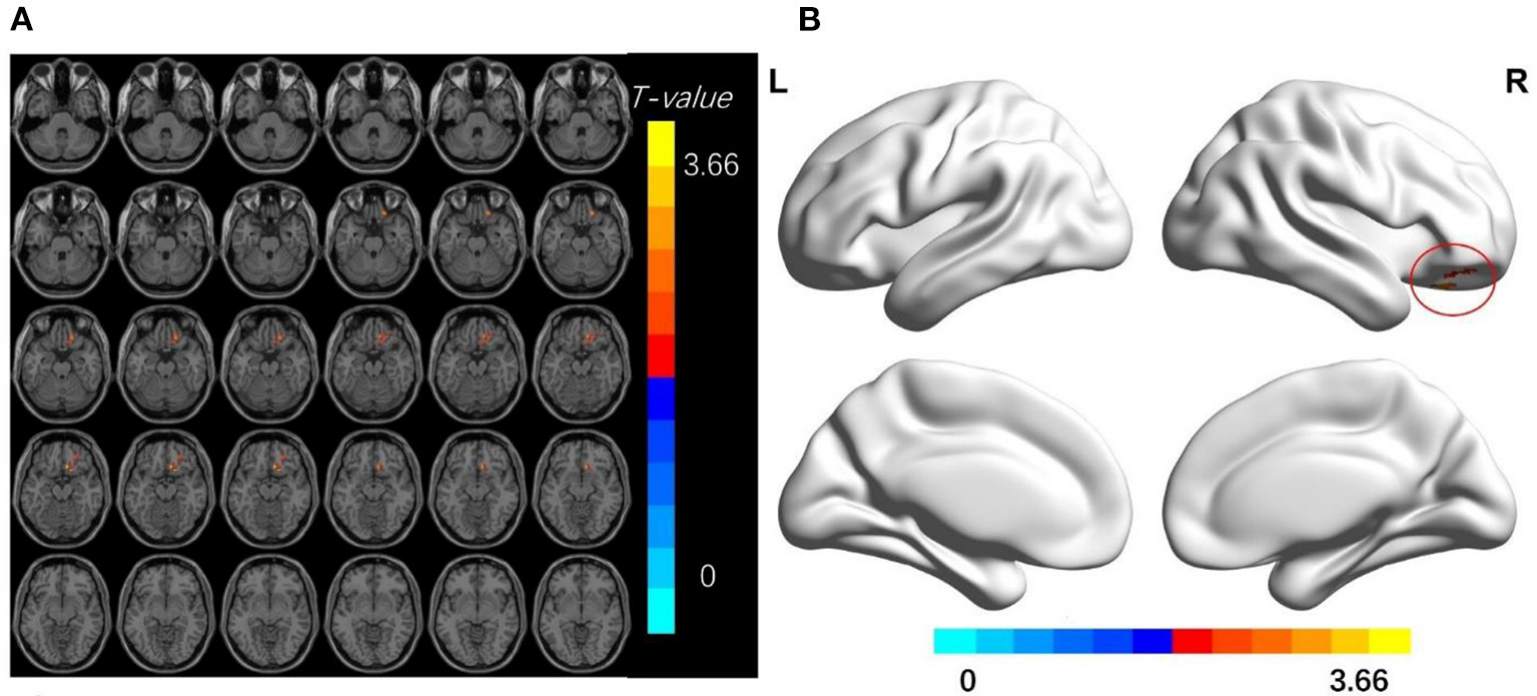
**(A,B)** The differences in dALFF between the TA patients and HCs (after GRF correction voxel-wise *p* < 0.01, cluster-wise *p* < 0.05, two-tailed). The color bar indicates the *t*-value. d-ALFF, dynamic low-frequency fluctuation amplitude; HCs, healthy controls; TA, toothache; MNI, Montreal Neurological Institute.

**Figure 3:**
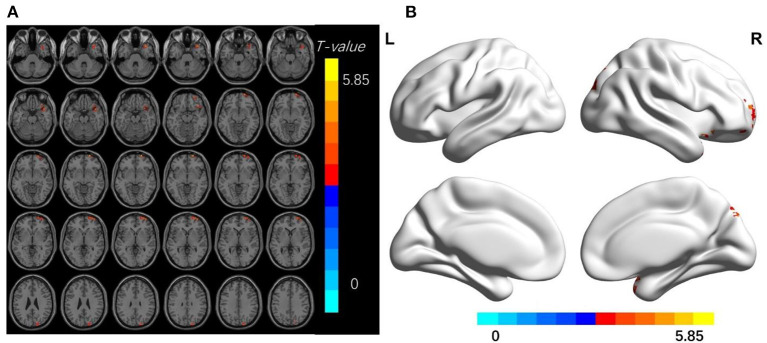
**(A,B)** The differences in dfALFF between the TA patients and HCs (after GRF correction voxel-wise *p* < 0.01, cluster-wise *p* < 0.05, two-tailed). The color bar indicates the *t*-value. d-ALFF, dynamic fractional low-frequency fluctuation amplitude; HCs, healthy controls; TA, toothache; MNI, Montreal Neurological Institute.

Compared with the HC group, TA patients had increased dReHo values in the bilateral middle frontal gyrus, right superior frontal gyrus, middle temporal gyrus, postcentral gyrus, left superior temporal gyrus, left precentral gyrus, precuneus, and angular gyrus; increased dALFf values in the right superior frontal gyrus, and increased dfALFF values in the right middle temporal gyrus, middle frontal gyrus, and superior occipital gyrus (*p* < 0.01, cluster level *P* < 0.05; Figures 1–3 and Tables 2–4).

**Table 2:** Brain regions with significant differences in dReHo between TA patients and HCs.

**Brian regions (AAL)**	**Hemisphere**	**Peak *T*-value**	**Voxels**	**Peak MNI coordinate (mm)**		
				**X**	**Y**	**Z**
Frontal _Sup	R	4.65	236	24	66	12
Temporal_Sup	L	4.21	101	−66	−51	−3
Frontal _Mid	L	3.72	93	−33	63	0
Precentral	L	4.22	225	−45	9	24
Temporal_Mid	R	3.87	201	24	30	33
Precuneus	L	4.96	445	3	−63	57
Postcentral	R	4.56	130	48	−45	42
Frontal _Mid	R	4.02	90	39	12	42
Angular	L	4.43	375	−30	−78	45

All clusters were reported with a Voxel-level threshold of *P* < 0.01, and cluster level *P* < 0.05, two-tailed, GRF corrected for. MNI, Montreal Neurological Institute; dReHo, Dynamic regional homogeneity.

**Table 3:** Brain regions with significant differences in dALFF between TA patients and HCs.

**Brian regions (AAL)**	**Hemisphere**	**Peak *T*-value**	**Voxels**	**Peak MNI coordinate (mm)**		
				**X**	**Y**	**Z**
Frontal _Sup	R	3.66	53	9	24	−15

All clusters were reported with a Voxel-level threshold of *P* < 0.01, and cluster level *P* < 0.05, two-tailed, GRF corrected for. MNI, Montreal Neurological Institute; dALFF, Dynamic low-frequency fluctuation amplitude.

**Table 4:** Brain regions with significant differences in dfALFF between TA patients and HCs.

**Brian regions (AAL)**	**Hemisphere**	**Peak *T*-value**	**Voxels**	**Peak MNI coordinate (mm)**		
				**X**	**Y**	**Z**
Temporal_Mid	R	4.58	80	36	21	−33
Frontal _Mid	R	5.85	79	21	69	−6
Occipital_Sup	R	4.18	63	21	−81	45

All clusters were reported with a Voxel-level threshold of *P* < 0.01, and cluster level *P* < 0.05, two-tailed, GRF corrected for. MNI, Montreal Neurological Institute; dfALFF, Dynamic fractional low-frequency fluctuation amplitude.

### Validation results

The validation analysis shows that the TA-related dynamic changes in the three local dynamic indicators are consistent with the main results when different window lengths are used. While keeping other computational parameters constant, the size of the window length was varied separately in our study to compare all results and increase their credibility. The results for different window lengths are shown in Supplementary Figures 1–3.

### Correlational analysis

**Figure 4:**
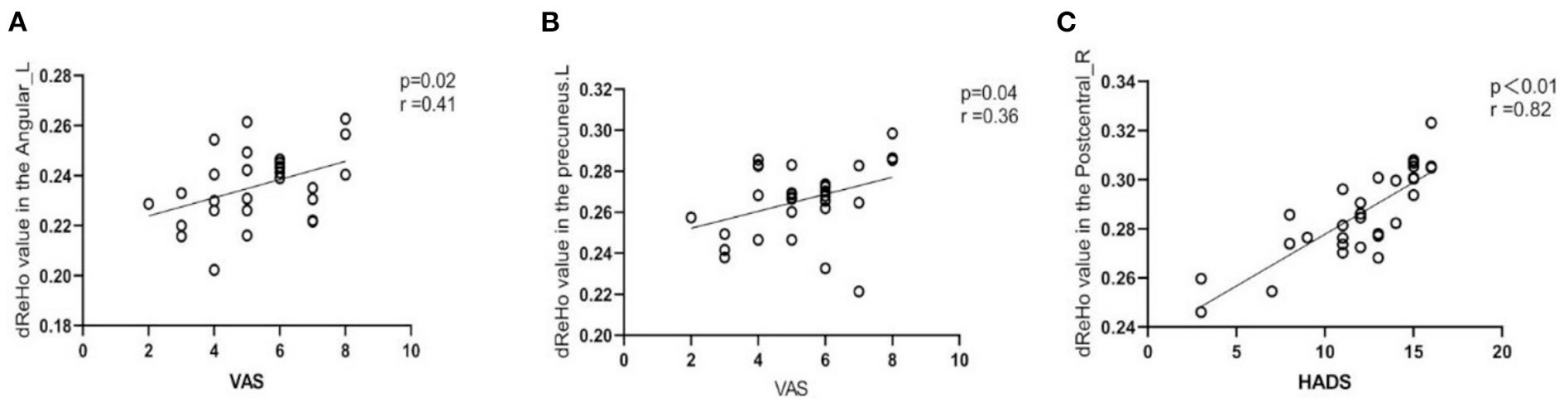
The correlation between local dynamic metrics in abnormal brain areas and clinical variables in patients with TA. **(A)** The left angular (*r* = 0.4 I and *P* = 0.02) dReHo values were significantly and positively correlated with the visual analog scale (VAS). **(B)** The left precuneus (*r* = 0.36 and *P* = 0.0.04) dReHo values were significantly and positively correlated with the visual analog scale (VAS). **(C)** The right postcentral gyrus (*r* = 0.82 and *p* < 0.01) dReHo value was significantly and positively correlated with the Hospital Anxiety Depression Scale score (HADS).

Pearson correlation analysis showed that the dReHo values of the left angular (Figure 4A) and left precuneus (Figure 4B) were positively correlated with VAS scores in the TA group. There was a positive correlation between dReHo and HADS scores in the right posterior central gyrus of the TA group (Figure 4C). The remaining abnormally active brain regions, dALFF values, and dfALFF values were not significantly correlated with VAS and HADS scores (*P* > 0.05).

## Discussion

**Figure 5:**
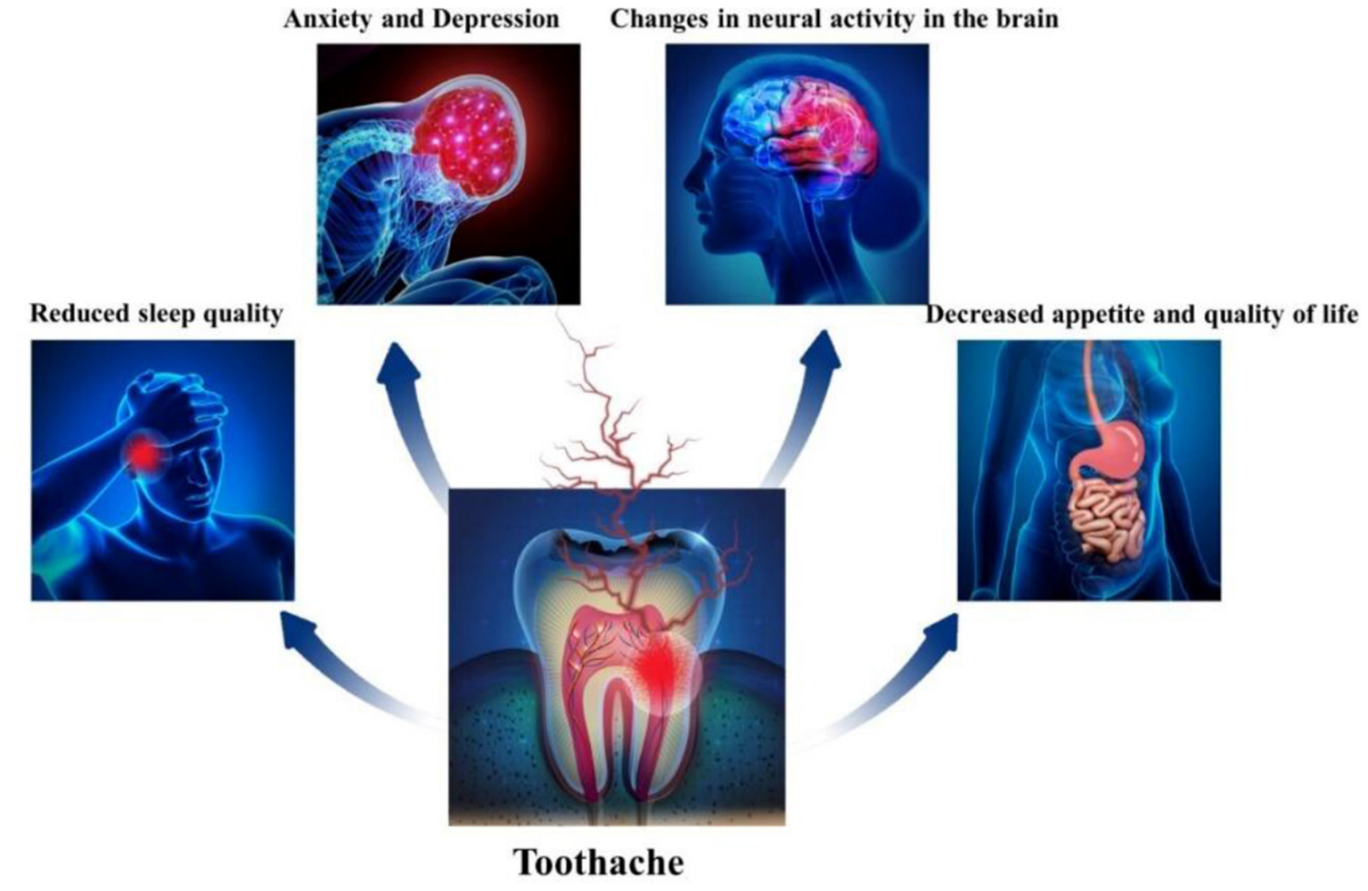
A demonstration chart of the impact of toothache on life.

Pain is a common, unpleasant, subjective experience, and is one of the main reasons for visiting a healthcare facility, with approximately 40% of people suffering from different types of pain (37). Pain processing does not occur in a single brain region but is widely distributed in the cortical and subcortical network called the “pain matrix” (38, 39), which influences cognitive functions, such as memory, attention, and executive functions. Toothache is transmitted to the trigeminal ganglion by stimulating nociceptors, and the trigeminal nerve projects to neurons at higher levels of the brain through the thalamus, causing corresponding changes in the cerebral cortex (40). Also toothache can have a great impact on our daily life (Figure 5). rs-fMRI has been widely used to study spontaneous brain activity in pain-related disorders, such as lower back pain, migraine, trigeminal neuralgia, and postherpetic neuropathic pain. ReHo, ALFF, and fALFF are relevant metrics for studying changes in local spontaneous brain activity, which are measured under the assumption that the BOLD signal is stationary during the fMRI scan. However, studies show that the brain is a highly active system, and the BOLD signal constantly changes with the time of the scan (41). dReHO, dALFF, and dfALFF can reflect the change in amplitude of local spontaneous brain activity over time and can better reflect the change in spontaneous brain function compared with static local indexes. In this study, we used three rs-fMRI local dynamic indices to study alterations in intrinsic brain activity in patients with TA. Patients with TA had increased dReHo values in the bilateral middle frontal gyrus, right superior frontal gyrus, middle temporal gyrus, postcentral gyrus, left superior temporal gyrus, left precentral gyrus, precuneus, and angular gyrus; increased dALFf values in the right superior frontal gyrus; and increased dfALFF values in the right middle temporal gyrus, middle frontal gyrus, and superior occipital gyrus. These results provide more information to study the neural mechanisms of altered brain function caused by TA in the expectation of finding neuroimaging markers that are useful for TA diagnosis and clinical work.

ReHo primarily measures the temporal synchrony of regional neural activity between spatially adjacent regions (22), and dReHo reflects the change in similarity between the time series of a specific voxel and its nearest neighbors (42, 43). Patients with TA had increased dReHo values in the Left superior temporal gyrus, middle frontal gyrus, precentral gyrus, precuneus, angular gyrus, right superior frontal gyrus, middle temporal gyrus, postcentral gyrus, middle frontal gyrus. The middle frontal gyrus is thought to be related to the dispose of pain, and in more pain-related studies, abnormal changes in the middle frontal gyrus, such as migraine, postherpetic neuralgia, and lower back pain (44–46). Research have shown that the frontal cortex has the role of information integration, which can link pain and related behaviors, mainly involved in the integration and processing of pain information, and the frontal gyrus of TA patients is activated, which is related to the processing and integration of pain information. The superior temporal gyrus is associated with the management of pain, while the temporal lobe is thought to be associated with language processing (47), and damage to this brain region may lead to language disorders, and the activation of the superior temporal gyrus in TA patients is caused by increased pain processing caused by TA, as well as some impact on language processing. The precentral gyrus is part of the first motor cortex (M1) and is involved in internally generated motor planning and in pain anticipation (48, 49). M1 is also a frequently abnormally altered brain region in pain-related studies, and activation of the anterior central gyrus in TA patients may be associated with covering the affected side of the face with the patient's hand during toothache, and may also be associated with causing anxiety in patients. The angular gyrus is considered to be a common hub connecting several networks of the “pain matrix,” participating in higher-order brain functions and integrating bottom-up multi-sensory information and top-down cognitive prediction (50); the angular gyrus was activated in this study, which may be related to the central transmission of pain. The precuneus is considered the central node of the DMN and plays a key role in the DMN. Some studies have shown that the precuneus is an active region during the pain awareness window, which continues to be active after the pain-induced voluntary movement, and plays a role in consolidating immediate pain perception and developing memory (39). Activation of the precuneus in TA patients is associated with the presence of insomnia and anxiety in TA patients, and may also be related to TA, leading to the formation of pain memories. The postcentral gyrus is also known as the primary sensory cortex (S1), which is part of the pain matrix (38). At the same time, S1 plays an important role in pain elicitation and processing, a conclusion confirmed in primate studies (51, 52). It has also been shown to be activated in many acute experimental pain studies, and S1 was activated in TA patients in this study, in relation to S1 to pain processing and the projection of pain transmission pathways in S1.

ALFF reflects the power of the brain in the frequency range of 0.01–0.08 Hz, and its measurement of signal intensity in low-frequency oscillations of local spontaneous neural activity can reflect the intensity of spontaneous local fluctuations in the brain (23). DALFF reflects the dynamic change of ALFF (41). In the present study, dALFF values were increased in the right superior frontal gyrus. Previous studies have demonstrated that the superior frontal gyrus is associated with the integration and processing of pain signals (53, 54), and abnormal alterations in the superior frontal gyrus occur in many pain-related disorders, such as tension migraine (54), trigeminal neuralgia (55), and lower back pain (56), etc. In the present study, it was shown that increased dALFF in the right superior frontal gyrus in TA patients may be associated with improved pain perception and modulation in TA patients.

The ratio of the amplitude in the low-frequency range of the fALFF to the total amplitude in the full frequency range reveals the relative effect of a specific low frequency over the entire frequency range (24). dfALFF reflects the dynamic changes in fALFF and reveals the characteristics of temporal and low-frequency oscillatory changes in the power of spontaneous brain activity (37). In this study, TA patients had increased dfALFF values in the right middle temporal, frontal middle, and right superior occipital gyri. Studies have shown that the middle temporal gyrus is associated with emotion modulation and executive function and that TA patients experience irritability, anxiety, and other emotions that may be related to the activation of the middle temporal gyrus. The middle frontal gyrus, which includes the motor cortex and supplementary motor areas, is associated with pain processing, and the frontal cortex receives sensory information from pain-related cortical areas and plays an important role in the cognitive regulation of pain (6). Abnormal activation of the middle frontal gyrus in TA patients is mainly associated with altered pain transmission and processing. The occipital lobe is mainly associated with visual stimuli, awareness of facial emotions, and working memory (57, 58); increased dfALFF in the superior occipital gyrus in patients with TA may be related to the activation of visual stimuli.

Correlation analysis showed that dReHO values of left angular gyrus and left precuneus were positively correlated with VAS scores in TA group. Angular gyrus is a common center connecting multiple networks of “pain matrix,” and precuneus is the central node of DMN. We hypothesized that pain would cause changes in “pain matrix” and DMN, and the more severe the pain, the more obvious the activation., The dReHo values of the right postcentral gyrus was positively correlated with the HADS score. The postcentral gyrus is the primary sensory cortex, part of the “pain matrix,” and is involved in the processing of pain. There is a correlation between pain and anxiety and depression, and the stronger the activation of the postcentral gyrus, the more pronounced the degree of anxiety and depression.

## Conclusion

In conclusion, patients with TA have abnormal alterations in pain-processing brain regions, emotion-processing brain regions, and pain-cognitive modulation brain regions, and these brain regions and correlate with VAS and HADS scores, suggesting that TA causes abnormal cognitive and emotional alterations, which helps to reveal the underlying neuropathological mechanisms.

## Limitation

Although this study revealed dynamic local metric changes in patients with TA, it had some limitations. First, only 30 TA patients were included in this study, which is a small sample size, and this study was a single-center study. We will expand the sample size in subsequent studies. Second, this study was not conducted longitudinally, but mainly as a cross-sectional study. In the future, we will conduct a longitudinal study to observe the treatment methods of TA and the changes in brain function at each treatment stage and compare the treatment effects of each treatment method and each stage. Third, the inclusion criteria for TA patients were not strict, did not further distinguish between acute and chronic TA, and did not clearly distinguish between odontogenic and non-odontogenic pain, In the future, TA will be further refined and classified. Finally, the remaining temporomandibular joint (TMJ)-related problems and toothache caused by bruxism were not included in this study and will be added in later studies to make the study more enriched and reliable. Despite the above and limitations of our study, the pathogenesis of TA can still be considered to be related to abnormalities in specific areas of the brain, which can help to find an objective and reliable neurobiological imaging marker of TA as well as to further understand the effects and modulation of pain on consciousness and cognition.

## Data availability statement

The raw data supporting the conclusions of this article will be made available by the authors, without undue reservation.

## Ethics statement

The studies involving human participants were reviewed and approved by the Medicinal Morality Council of Nanchang University's First Affiliated Hospital. The patients/participants provided their written informed consent to participate in this study. Written informed consent was obtained from the individual(s) for the publication of any potentially identifiable images or data included in this article.

## Author contributions

YH guided and designed the MRI experiment, besides, they reviewed and revised the manuscript. XT and BL analyzed the resting-state fMRI data. MW and GX analyzed and discussed the ideas of the manuscript. MW organized the results and wrote the manuscript. TW and XZ helped with statistics and graphing. YZ and XL collected resting fMRI data and applied for the ethics. All authors contributed to the article and approved the submitted version.
