# Supplementary figures and images for: Dynamic local metrics changes in patients with toothache: A resting-state functional magnetic resonance imaging study
Source: Front Neurol. 2022 Dec 12;13:1077432. doi: 10.3389/fneur.2022.1077432 (PMC9790921; doi:10.3389/fneur.2022.1077432)

Supplementary Materials


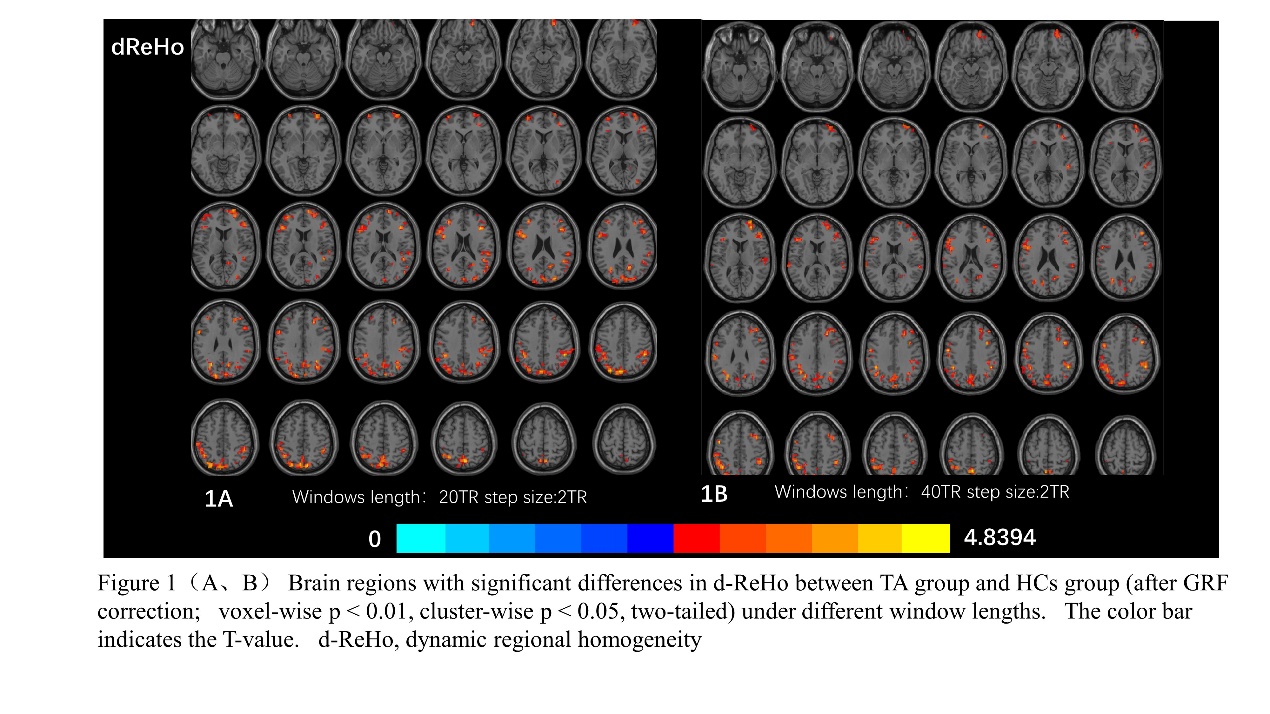

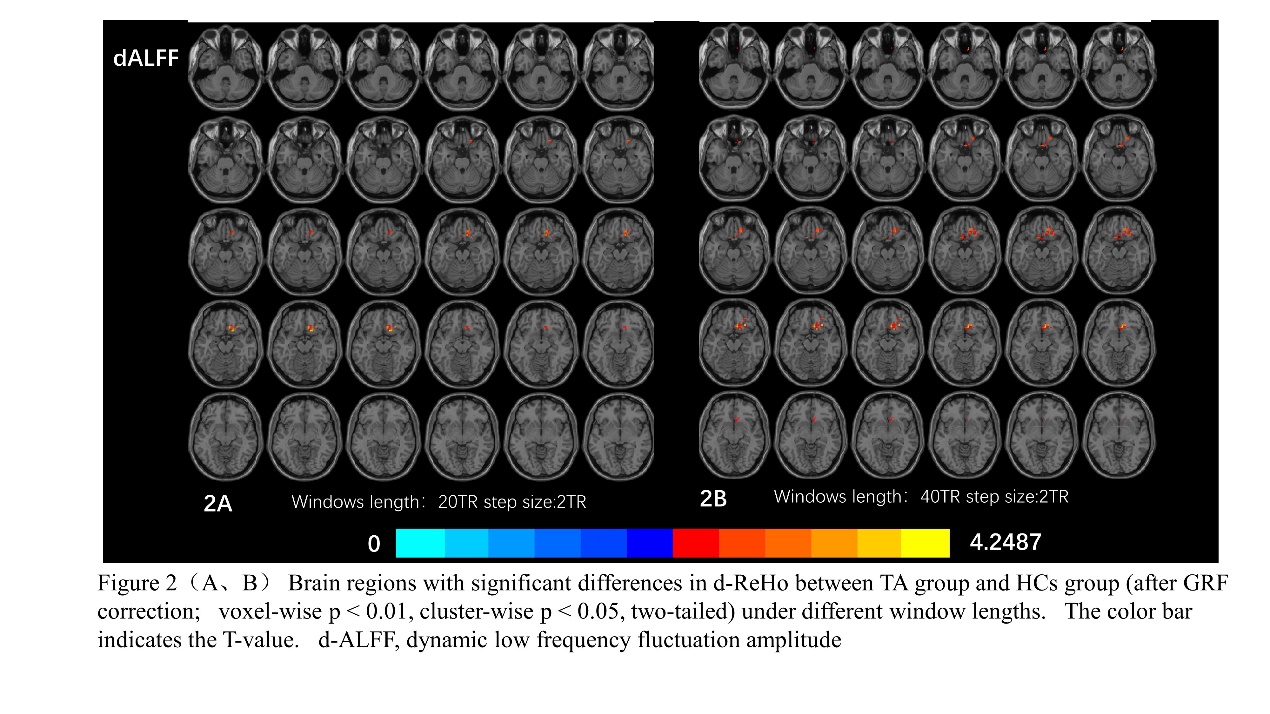

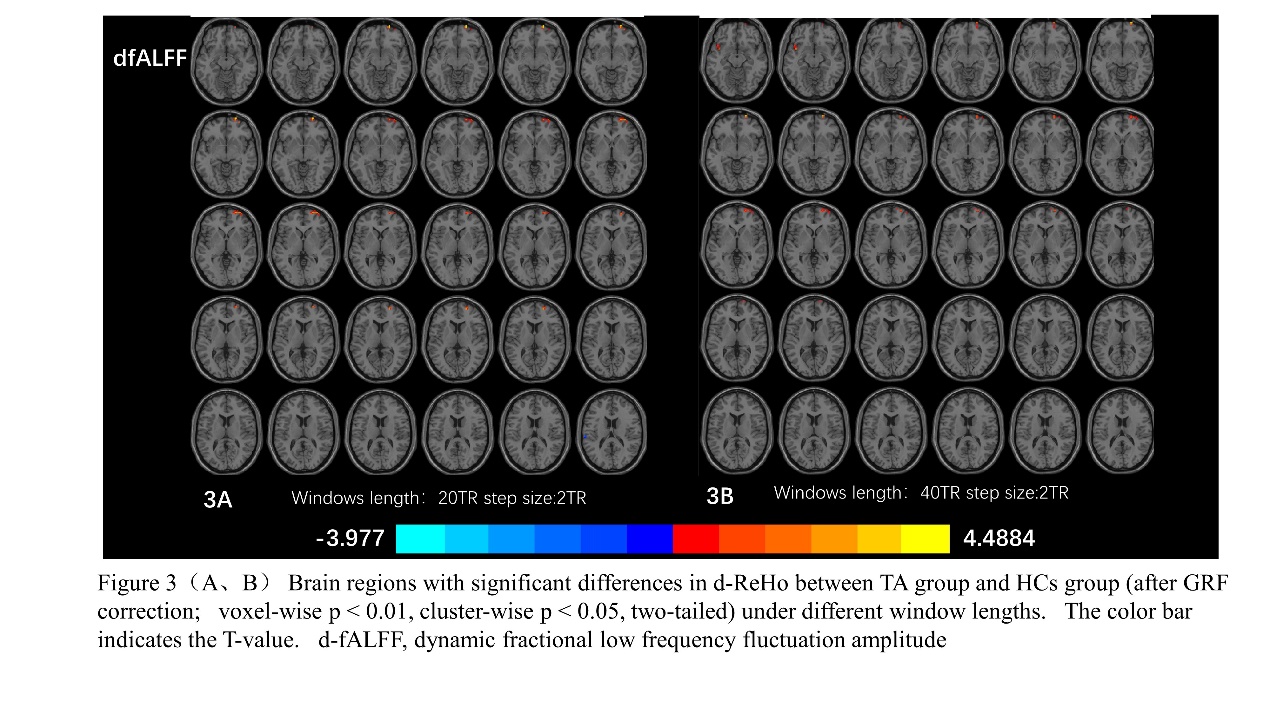

Supplement: Supplementary file 1 [file Data_Sheet_1.docx]
